# Synergistic application of calcium oxide nanoparticles and farmyard manure induces cadmium tolerance in mung bean (*Vigna radiata* L.) by influencing physiological and biochemical parameters

**DOI:** 10.1371/journal.pone.0282531

**Published:** 2023-03-02

**Authors:** Muhammad Waqas Mazhar, Muhammad Ishtiaq, Mehwish Maqbool, Muhammad Ajaib, Iqbal Hussain, Tanveer Hussain, Abida Parveen, Sumaira Thind, Tauqeer Sardar, Raheel Akram, Muhammad Azeem, Alia Gul

**Affiliations:** 1 Department of Botany, Mirpur University of Science & Technology (MUST), Mirpur, (AJK), Pakistan; 2 Department of Botany, Government College University, Faisalabad, Pakistan; 3 Department of Science, University of Central Punjab, Lahore, Punjab, Pakistan; 4 Department of Biology, College of Science, University of Bahrain, Zallaq, Bahrain; 5 Department of Botany Hazara University, Mansehra, KP, Pakistan; Central Research Institute for Dryland Agriculture, INDIA

## Abstract

Mung bean (*Vigna radiata* L.) grown under heavy metals such as cadmium stress shows poor growth patterns and yield attributes which can be extenuated by the application of calcium and organic manure to the contaminated soil. The present study was designed to decipher the calcium oxide nanoparticles and farmyard manure-induced Cd stress tolerance through improvement in physiological and biochemical attributes of mung bean plants. A pot experiment was conducted by defining appropriate positive and negative controls under differential soil treatments with farmyard manure (1% and 2%) and calcium oxide nanoparticles (0, 5, 10, and 20 mg/L). Root treatment of 20 mg/L calcium oxide nanoparticles (CaONPs) and 2% farmyard manure (FM) reduced the cadmium acquisition from the soil and improved growth in terms of plant height by 27.4% compared to positive control under Cd stress. The same treatment improved shoot vitamin C (ascorbic acid) contents by 35% and functioning of antioxidant enzymes catalase and phenyl ammonia lyase by 16% and 51%, respectively and the levels of malondialdehyde and hydrogen peroxide decreased by 57% and 42%, respectively with the application of 20 mg/L CaONPs and 2% of FM. The gas exchange parameters such as stomata conductance and leaf net transpiration rate were improved due to FM mediated better availability of water. The FM improved soil nutrient contents and friendly biota culminating in good yields. Overall, 2% FM and 20 mg/L CaONPs proved as the best treatment to reduce cadmium toxicity. The growth, yield, and crop performance in terms of physiological and biochemical attributes can be improved by the application of CaONPs and FM under the heavy metal stress.

## Introduction

Mung bean [*Vigna radiata* (L.) Wilczek] is a top legume crop grown under warm season climates. The crop is highly enriched with nutrients and phytochemicals [1]. Mung requires less water and is cultivated in arid zones worldwide. The yield and production of mung beans have decreased worldwide due to heavy metal accumulation. The growth, production, yield, and protein bioavailability of mung bean has decreased due to contaminated agricultural soils with accumulation of heavy metals [2].

Cadmium (Cd) is one of the highly toxic metals to living organisms. Present developments in agriculture and industry have increased concentrations of Cd in the croplands. Due to its high mobility in polluted soils, Cd is highly toxic to the plants and environment [3]. Cadmium contamination results in enzymatic inhibition, nutrient deficiency, and free radical accumulation in the mung bean plants which leads to yield limitations and a decline in crop performance. Cd has a higher rate of exchange with Calcium (Ca) due to similar charges and ionic radius. Due to this resemblance, Cd is easily acquired by the roots of the mung bean plants and it results in chlorosis and stunted growth decreasing the yield and production. Higher levels of soil rhizosphere Cd result in poor stomatal conductance, reduction in net photosynthesis, and reduced transpiration in the mung bean plants. Cd toxicity increases the concentration of reactive oxygen species (ROS). Higher levels of Cd induce poor nutrient acquisition patterns in mung plants targeting their growth and agronomic performance [4]. In response, plants have employed many adaptive methods to counteract and minimize the harmful effects of Cd. The main detoxifying processes are Cd build-up and exclusion in particular plant sections. With the aid of signalling pathways that control survival and growth under Cd stress, plants can also adapt to Cd toxicity.

Calcium (Ca) is a macronutrient involved in physiological wellbeing in plants. Ca performs several structural and physiological roles including signalling and sensing roles towards environmental stimuli [5]. Increased cytosolic contents of Ca^+2^ is important for the biosynthesis of cell wall precursors. Ca forms complexes with the cell membrane lipids and thus it stabilizes the membrane structure. Ca is required by the seed plants for elongation of their pollen tubes and it is important for the development of root hairs. Additionally, Ca acts as important signalling metal in cell cycle regulation events. Ca is involved in the activation of ion channels in response to environmental stimuli and thus it is a versatile signalling molecule [6]. Over the years, nanotechnology has become a promising tool to achieve agricultural sustainability. Calcium oxide nanoparticles (CaONPs) are being utilized nowadays in agricultural research with promising results. Recently, researchers have reported the promising applications of CaONPs in several crop species including *Cicer arietinum* [7], *Cucurbita pepo* [8] and *Oryza sativa* [9]. Results of these studies have suggested that CaONPs nanoparticles might be promising candidates for reducing heavy metal stress, drought stress, and biotic stress, and thus CaONPs might be helpful in food security. Soil organic amendments are important to build soil structure, improve the nutrient profile and increase fertility in croplands. Farmyard manure (FM) is a soil organic amendment that provides the soils with major nutrients such as Ca, S, N, K, P and Mg. FM is an excellent source of some micronutrients such as Fe, Mn, Cu and Zn [10]. All of these nutrients are crucial to plant survival, growth and completion of the life cycle. FM improves soil structure, porosity and physiochemical traits and thus FM application to soil induces tolerance to environmental stress such as heavy metal stress [11].

As a trace metal, Cd is generally safe at concentrations up to 0.1 mg/kg. Any metallic chemical compound that has a relatively high density and is dangerous and toxic even at low concentrations is referred to as a trace metal. The majority of inorganic contaminants in soils do not degrade chemically or microbiologically and their concentration remains in the soil for a very long time after application. However, the bioavailability and chemical forms of metal contaminants may fluctuate. By reducing leaf relative water content, stomatal conductance and transpiration, exposure to Cd in soil causes osmotic stress in plants which causes physiological harm to the plants. Due to the role of Ca in signalling pathways that regulate survival of plants under heavy metal stress, present research was designed to study its impact along with FM in mitigation of heavy metal stress on the mung bean plants which are sensitive to heavy metals such as Cd. Scarce and sporadic work has been reported on CaONPs application on mung bean plants. The objective of the present research was to comprehend the beneficial roles of CaONPs and FM application on mung bean plants in mitigating Cd stress. The role of Ca in reducing the uptake of Cd and inducing tolerance to Cd stress was investigated by studying antioxidant defence responses, yield, growth and cytosolic non-enzymatic antioxidants of mung bean plants. The hypothesis of the present research was that synergistic application of FM and CaONPs might reduce the bioavailability of Cd in mung bean plants and the application of these treatments improves the growth and agronomic traits of mung bean plants. This work will provide future directions to figure out the underlying molecular mechanisms involved in the mitigation of heavy metals in various plants as there is a paucity of knowledge regarding the mechanisms involved in the amelioration of cadmium toxicity.

## Materials and methods

### Experimental design

The pot experimental trial was run from April to June 2022. The used plastic pots had 7′′ x 6′′ length and breadth measurements. The pots were stored in the Government Graduate College Sarai Alamgir experimental research area (32.8849090, 73.7571688) District Gujrat, Pakistan. Seeds of Vigna radiata cv. Azri-2006 were obtained for experimental trial from a local agricultural goods market. The experimental soil was collected from a farmland area near the site of experiment. The physicochemical properties of the experimental soils were appraised following Davis and Freitas [12] and have been presented in the Table 1.

**Table 1:** Physio-chemical attributes of the experimental soil used for cultivation of mung bean plants.

Soil Character	Recorded value
Clay	64%
Sand	23%
Silt	13%
Saturation percentage	31%
pH	8
Electrical conductivity	2.2 dSm^-1^
Available phosphorus	5.5 mg Kg^-1^
Available potassium	178 mg Kg^-1^
Available calcium	118 mg Kg^-1^
Nitrogen (NO^-3)^	6.4 mg Kg^-1^
Nitrogen (NH^+4^)	3.1 mg Kg^-1^
Organic matter	0.78%
Soil intrinsic Cadmium	3.08 mg Kg^-1^

**Fig 1:**
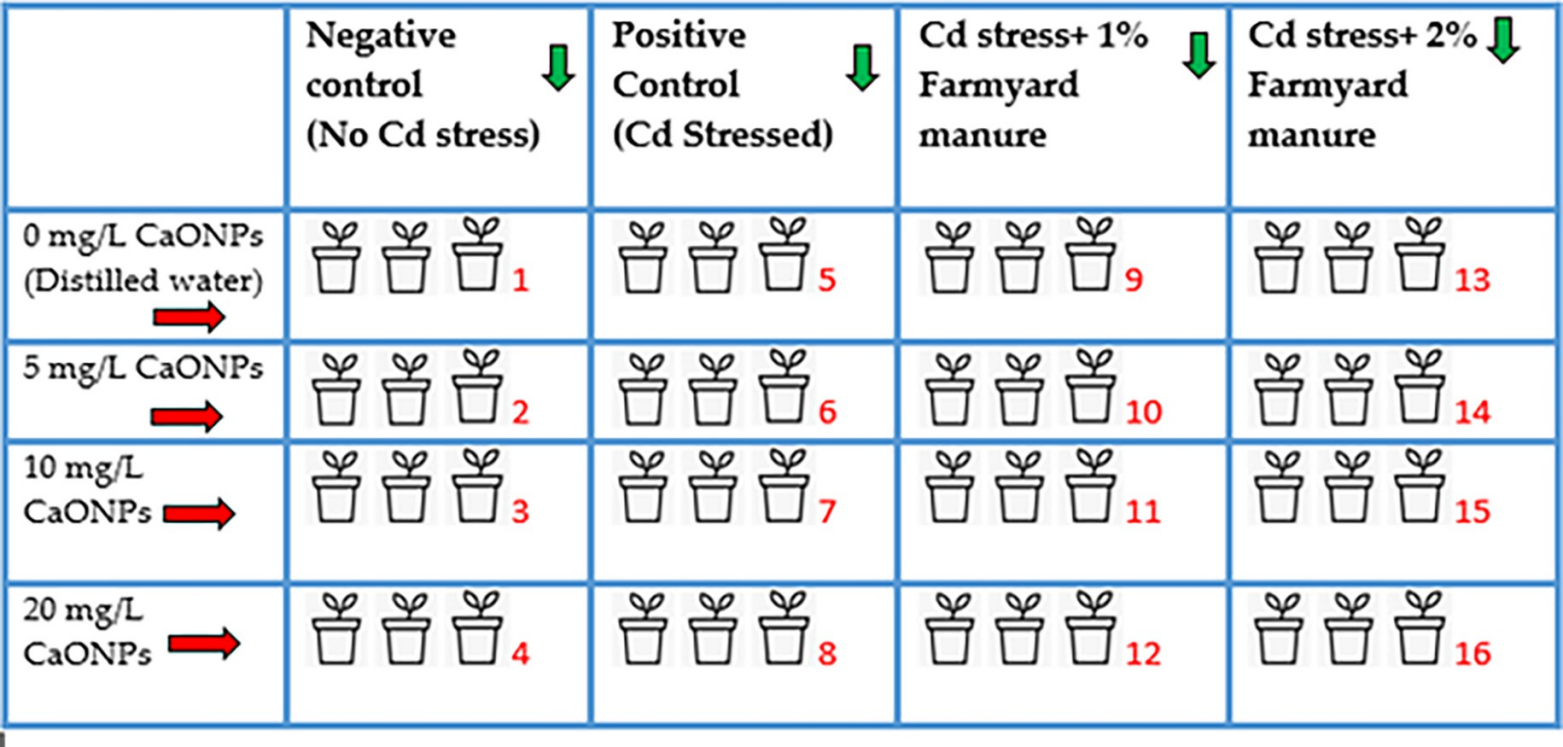
Graphical representation of the treatment plan and experimental setup layout for the current study. A total of 16 treatments were used indicated by number in red fonts. Arrows in green represent treatments in columns and arrows in red represent treatments in rows. A total of 48 pots were the part of the experimental setup.

The CaONPs were obtained from American Elements (Product No. CA-OX-02-NP). The nanoparticles were 20–80 nm in size distribution ranges averaging 50 nm. The specific surface area ranged from 15–50 m^2^/g averaging 33 m^2^/g. The density of CaONPs was 3.3 g/cm^3^ with a purity percentage of 97.5%. To make treatment concentration of 5, 10 and 20 mg/L, the CaO nano-powder was weighed at 5, 10 and 20 mg and was dissolved into a litre of water. The mixtures were ultrasonicated to achieve uniform dispersions [13]. To find the ideal Cd levels, a preliminary experiment was run using various concentrations of cadmium chloride (0, 50, 100, 150, and 200 M). Based on the findings of this experiment, the 150 M Cd stress which inhibited growth and germination of mung bean by 50% was chosen for the current work. Cadmium chloride 150 μM salt was mixed to the soil within the pots except the pots with negative control for the purpose of Cd stress after three weeks of seed germination in the pots. The trial was conducted for 75 days in accordance with Khan *et al*., [14]. In the test pots, the seeds were sowed after being surface sterilised (7 seeds per pot). The plant emergence took place in the experimental pots. After three weeks of germination the soil near the roots of plants was carefully removed and a single treatment with of varying concentrations of CaONPs 5, 10 and 20 mg/L was supplied in the soil near the root zone after two hours of Cd treatment with irrigation water. A total of 4 irrigations were supplied to the experimental mug bean plants. First irrigation was performed at the time of sowing, second irrigation was performed along with root treatments with CaONPs at the trifoliate leaf stage (three weeks after germination), third irrigation was performed at flowering stage and last irrigation was performed at pod filling stage. The pot experimental design used a totally random approach. A total of 48 pots with 16 treatments (in triplicates) were employed. As negative controls (NC) for the experiment, plants with treatments 1, 2, 3 and 4 were grown on soil without Cd salt and given root treatments with distilled water (DW) as control, 5 mg/L of CaONPs, 10 mg/L of CaONPs and 20 mg/L of CaONPs, respectively. On cadmium contaminated soil (Cd), plants with treatments 5, 6, 7 and 8 received root treatments with distilled water (DW), 5 mg/L of CaONPs, and 10 mg/L and 20 mg/L CaONPs were used as the experiment’s positive controls. Plants with treatments 9, 10, 11 and 12 received root treatments with distilled water (DW), 5 mg/L of CaONPs, 10 mg/L of CaONPs, and 20 mg/L of CaONPs, respectively on Cd contaminated soil that had been supplemented with 1% FM (Cd+1% FM). Plants with treatments 13, 14, 15 and 16 received root treatments with distilled water (DW), 5 mg/L of CaONPs, 10 mg/L of CaONPs, and 20 mg/L of CaONPs, respectively, on Cd stressed soil that had been modified with 2% FM (Cd+2% FM) (Fig 1). The composition of FM has been presented in Table 2. The experimental space was equipped with two data loggers that were placed there to keep track of the temperature and humidity levels. A relative humidity of 73% and a mean temperature of 38.5°C were noted.

**Table 2:** Physiochemical attributes of farmyard manure used in the experimental soil.

Character	Composition
pH	6.8
Electrical conductivity	4.2 dSm^-1^
Nitrogen	8.6 g Kg^-1^
Phosphorus	4 g Kg^-1^
Potassium	8.4 g Kg^-1^
Calcium	76.7 g Kg^-1^
Magnesium	9 g Kg^-1^
Iron	5.7 g Kg^-1^
Manganese	321 mg Kg^-1^
Zinc	94 mg Kg^-1^
Copper	27 mg Kg^-1^
Organic carbon	112.2 g Kg^-1^
Intrinsic Cadmium	0.8 mg Kg^-1^

Data on root and shoot cadmium acquisition along with growth and yield parameters was collected on maturity i.e., 75 days old. The data on biochemical parameters such as malondialdehyde, hydrogen peroxide, antioxidant enzymes, vitamins and total chlorophyll contents was observed at flowering stage (37 days after germination) soon after third irrigation. The data on physiological attributes such as gas exchange parameters and stomatal conductance was also taken 37 days post germination.

### Growth and yield traits

The observation were made on plant height and number of branches of the mung bean for the purpose of recording growth attribute. To make a yield profile, observations on number of pods per plant, pod weights, seed count per pod and thousand seeds weight were recorded [15].

### Malondialdehyde and hydrogen peroxide contents

The MDA’s contents were evaluated in accordance with Cakmak and Horst’s procedure [16]. About 0.1g of leaf from each of the three replicates was homogenised in 5 mL of 0.1 percent TCA to determine the hydrogen peroxide values. The ice bath was utilised to keep the environment chilled while grinding. After grinding, centrifugation was carried out for 5 minutes at a speed of 12000 rpm. To 0.5 mL of phosphate buffer (pH 7.21), the resulting supernatant was added. Before reading the absorbance at 390 nm by Shimadzu-1900 UV spectrophotometer, 1 mL of 1M potassium iodide was added to the mixture and thoroughly mixed and the quantification was made by drawing a calibration curve prepared from a range of pure standards [17].

### Antioxidant enzymes assay

The catalase (CAT) activity was checked using the Chance and Maehly [18] procedure. Five minutes at 20°C were spent incubating the CAT reaction mixture (1 mL of enzyme extract + 1 mL of 0.01 M H_2_O_2_ + 0.1 M of 1 mL phosphate buffer having pH of 7.21). The addition of 10 mL of 1 percent H_2_SO_4_ stopped the reaction. When a reddish tint started to emerge, the mixture was titrated against 0.005 N KMnO_4_. The CAT estimation was based on the disappearance of H_2_O_2_ as basic phenomenon in reaction mixture measured as decrease in absorbance at 240 nm measured using Shimadzu-1900 UV spectrophotometer. The activity of PAL was estimated following Kim and Hwang [19]. Fresh leaf tissues were homogenized by using an ice-cold mortar and pestle in 100 mM phosphate buffer with 2 mM EDTA.

### Total chlorophyll assay

By using protocol devised by Arnon [20], the total chlorophyll contents of each experimental trial’s plants were assayed. Fresh leaves (0.25 g) were taken from each treatment and placed overnight chlorophyll extracted with 80% acetone at 4˚C. These extractions were centrifuged at 10,000 rpm for 5 min. The supernatant obtained was used for measuring absorption pattern at wavelength of 663, 645 and 480 nm by using spectrophotometer (Hitachi-U2001, Tokyo, Japan).

### Determination of ascorbic acid (AsA) and tocopherol contents

AsA’s contents were evaluated utilising the Mukharjee and Choudhari approach [21]. In this study, 0.25 g of mung bean leaves were ground in 10 mL of a TCA solution with a 6 percent concentration. A mixture of 4 mL of leaf extract and 2 mL of a Dinitrophenyl Hydrazine solution at 2% was used. One drop of 10% alcoholic thiourea was added to the mixture. After 20 minutes of heating, the mixture was cooled to room temperature. About 80 percent of H_2_SO_4_ (V/V) was combined in 5 mL of cooled down solution at 0°C. The absorbance ratio of the combination solution was measured at 530 nm by using a spectrophotometer (Shimadzu-1900 UV spectrophotometer). A standard curve that was created using various AsA standards allowed for the calculation of the AsA concentration in the extracted leaf sample.

Leaf total tocopherol contents were measured using a modified version of the Bakers [22], technique. Each sample’s fresh leaf material (0.5 g) was homogenised in 10 mL of a 2:1.6 (v/v) petroleum ether and ethanol solution before being centrifuged at 10,000 x g for 20 min. About 200 mL of 2-dipyridyl in ethanol (2%) were added to 1 mL of an aliquot and the mixture was thoroughly stirred and left in the dark for five minutes. The mixture was then given 4 mL of distilled and deionized water which was thoroughly mixed. The reading was obtained using a spectrophotometer (Shimadzu-1900 UV spectrophotometer) at 520 nm. Though using a standard curve created with known amounts of tocopherol, the total tocopherol content was determined.

### Recording gas exchange parameters

The gas exchange parameters such as stomatal conductance (*gs*), net CO_2_ assimilation (*A*) and transpiration rate (*E*) were examined by using a portable infra-red gas analyzer (IRGA) LCA-4 ADC (Analytical Development Company, Hoddesdon, England). The top-third fully formed leaf was used to estimate various gas exchange properties. The estimation was done between 9:00 and 12:00 in the morning. During the parameter estimate, the average light intensity ranged from 4.68 kWh/m^2^ /d to 5.54 kWh/m^2^ /d and average temperature was observed at 38.5°C [23].

### Cadmium determination in shoot and roots

As established by Abbas *et al*., [24], dry weights (0.5 g) of mung bean shoot and roots were digested in an acid mixture of HClO_4_ –HNO_3_ at a 1:3 ratio, respectively. A flame atomic absorption spectrophotometer (HITACHI Z-2000) for measuring atomic absorption recorded the amounts of Cd^+2^.

### Calcium determination in leaf and leaf relative water contents

For the estimation of calcium contents of mung bean plants leaf dry samples were powdered and digested with sulphuric acid following [23]. The quantification was performed using the atomic absorption spectrophotometer (AAS) (Hitachi. Model 7JO-8024, Tokyo, Japan).

Prior to start of each treatment; the leaf fresh samples were weighed. The leaves were then submerged for four hours in distilled water. Following that, leaves were wiped for surface water and weighed for turgid weights. After the leaves had been heated in an oven for 48 hours at 70°C, their dry weights were measured. The following equation was used to investigate leaf relative water content:

$$Leaf\;relative\;water\;content\;\%=[\frac{\left(Leaf\;fresh\;weight-Leaf\;dry\;weight\right)}{\left(Leaf\;turgid\;weight-Leaf\;dry\;weight\right)}]\times 100$$

### Statistical analysis

The data was analyzed using two-way analysis of variance research; first the data was recorded on a Microsoft Excel sheet using Co-STAT version 6.3 (developed by Cohort Software Berkley, CA, USA). XLSTAT version 2014 was used to construct the Pearson correlation matrix, heat map, and principal component analysis (Addinsoft, Paris France) [25].

## Results

### Root and shoot cadmium acquisition

**Fig 2:**
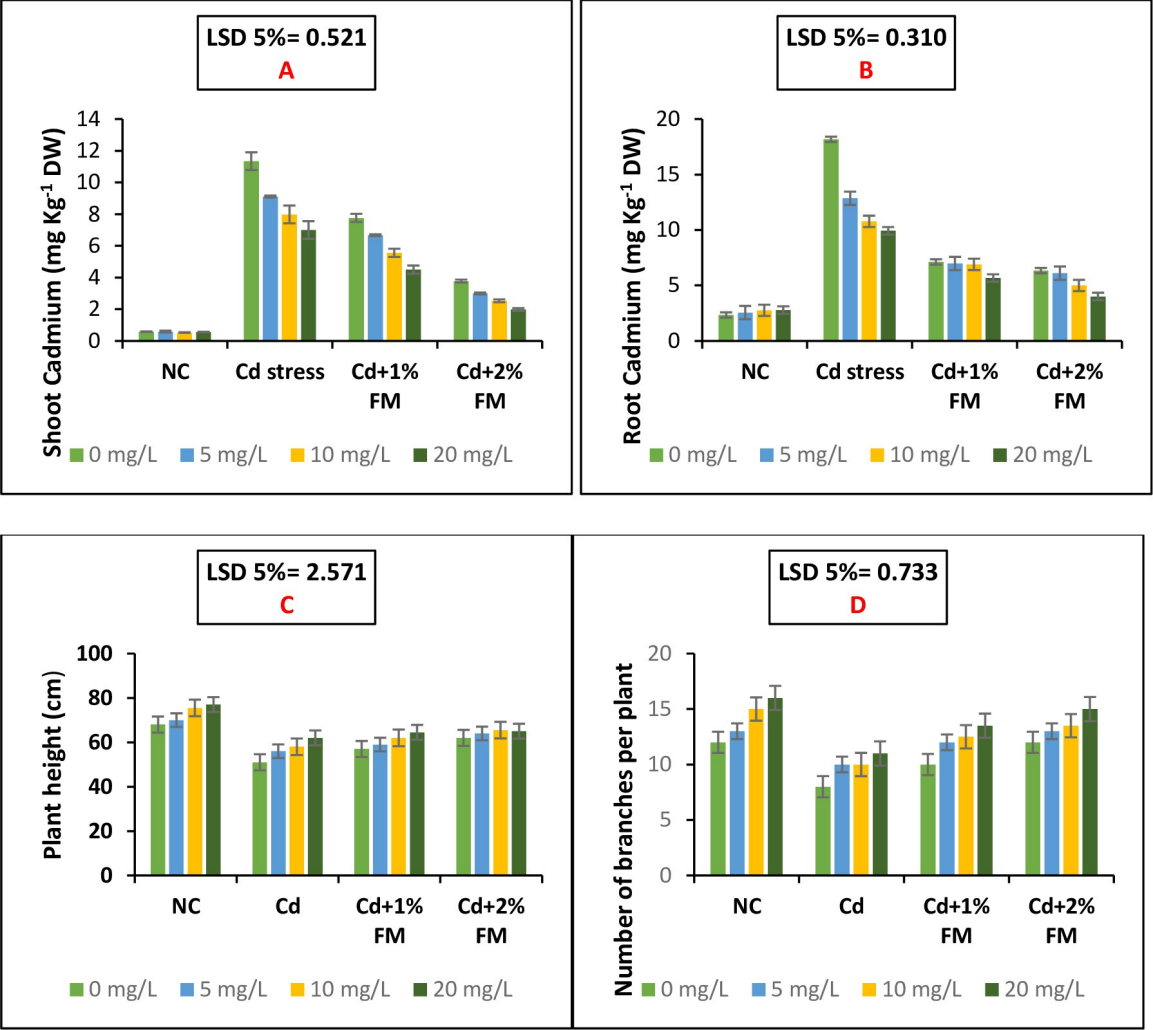
Shoot and root cadmium uptake and growth responses of mung bean plants grown under cadmium stress and treated with different levels of CaONPs and FM. On the x-axis, NC represents the negative control group; Cd represents the positive control group; Cd+1% FM represents the group treated with Cd and FM 1% and Cd+2% FM represents the group treated with Cd and FM 2%. In the legends 0, 5, 10, and 20 mg/L are root treatment doses of calcium oxide nanoparticles.

The synergistic application of CaONPs as root treatment and FM as soil amendment decreased the uptake of cadmium by the roots and shoots of mung bean plants. There was a linearly decreasing trend in root and shoot cadmium contents due to FM application and CaONPs. Addition of 2% FM and 20 mg/L CaONPs proved the most effective role in decreasing the cadmium acquisition from the soil by the mung bean plants through lowering cadmium acquisition in shoots and roots by 51.4% and 33.9%, respectively as compared to cadmium treated plants receiving 0 mg/L CaONPs and without farmyard manure applicated soil (Table 3; Fig 2A and 2B). A least significant difference of 0.521 and 0.310 was observed among the four main treatment groups in reducing Cd acquisition in shoots and roots of the experimental mung bean plants.

**Table 3:** Mean square and p values from the variables studied in the current experiment.

Dependent variable	Treatment effect	Degrees of freedom	Mean Square	P-Value
Shoot Cd	(i) Columns	3	71.786	0.000***
	(ii) Rows	3	0.416	0.043*
	Error	9	0.106	
Root Cd	(i) Columns	3	168.236	0.000***
	(ii) Rows	3	0.0239	0.7110 ns
	Error	9	0.037	
Plant Height	(i) Columns	3	181.765	0.000***
	(ii) Rows	3	42.378	0.000***
	Error	9	2.403	
Number of branches	(i) Columns	3	14.342	0.000***
	(ii) Rows	3	8.983	0.000***
	Error	9	0.298	
Total chlorophyll	(i) Columns	3	130.687	0.000***
	(ii) Rows	3	33.432	0.000***
	Error	9	1.182	
Leaf transpiration rate	(i) Columns	3	0.900	0.000***
	(ii) Rows	3	0.006	0.242 ns
	Error	9	0.003	
Net CO_2_ assimilation	(i) Columns	3	13350.242	0.000***
	(ii) Rows	3	12312.343	0.000***
	Error	9	57.506	
Stomata conductance	(i) Columns	3	21984.343	0.000***
	(ii) Rows	3	76.722	0.290 ns
	Error	9	16.452	
Ascorbic acid	(i) Columns	3	375.121	0.000***
	(ii) Rows	3	0.107	0.9517 ns
	Error	9	4.762	

*, ** and *** = significant at 0.05, 0.01, and 0.001 levels, respectively. ns; non-significant

### Growth attributes as influenced by the CaONPs and FM application

Data on the height and number of branches of mung bean plants have been presented in Fig 2C and 2D. The height and number of branches of the mung bean plants were significantly reduced upon imposition of cadmium toxicity. CaONPs treatment improved plant height and the number of branches. The increasing concentration of CaONPs increasingly affected the height and number of branches. Under the application of CaONPs, the FM effectively improved the plant height and number of branches and effectively mitigated the cadmium toxicity (Fig 2C and 2D). Addition of 2% FM and 20 mg/L CaONPs in the soil proved the best treatment in improving the height by 27.4% and number of branches of mung bean plants by 61% compared to the plants under cadmium application receiving 0 mg/L CaONPs and without farmyard manure applicated soil. A least significant difference of 2.571 and 0.733 was observed regarding plant height and number of branches among the four major treatment groups as shown in Fig 2C and 2D, respectively.

### Efficacy of synergistic application of CaONPs and FM in lowering the examined stress indicators and boosting the activities of antioxidant enzymes

**Fig 3:**
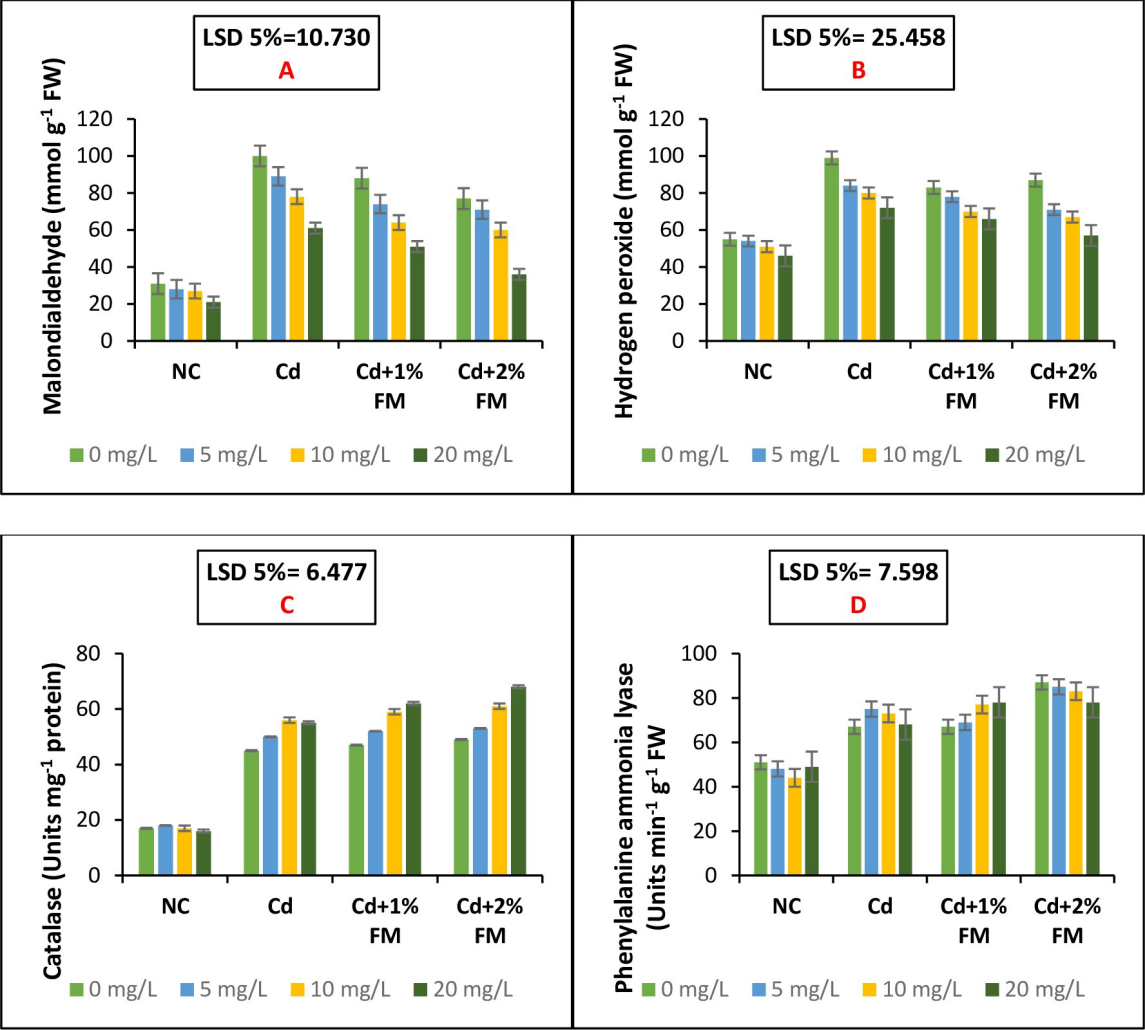
Levels of stress indicators (malondialdehyde and hydrogen peroxide) and antioxidant enzymes (CAT and PAL) in the mung bean plants grown in cadmium stress and treated with CaONPs and FM. On the x-axis, NC represents the negative control group; Cd represents the positive control group; Cd+1% FM represents the group treated with Cd and FM 1% and Cd+2% FM represents the group treated with Cd and FM 2%. In the legends 0, 5, 10, and 20 mg/L are root treatment doses of calcium oxide nanoparticles.

The imposition of cadmium stress on the experimental mung bean plants significantly enhanced the leaf malondialdehyde contents (Fig 3A). Similarly, the assayed reactive oxygen species in the form of hydrogen peroxide increased due to cadmium stress (Fig 3B). Application of CaONPs resulted in lowering of hydrogen peroxide contents and MDA accumulation. The combined application of CaONPs and FM effectively lowered the cadmium toxicity as MDA and hydrogen peroxide contents significantly declined due to applied treatments. About 2% FM and root treatment with 20 mg/L CaONPs proved the best in lowering hydrogen peroxide contents (42.2%) and MDA (64%) accumulation in mung bean plants grown under cadmium stress. Activities of antioxidant enzymes phenylalanine ammonia-lyase (PAL) and catalase (CAT) were assayed and the data has been presented in Fig 3C and 3D. Under cadmium stress, the functioning of PAL and CAT was enhanced significantly compared to the negative control. The application of CaONPs further improved the activities of these enzymes as the combined application of CaONPs and FM (20 mg/L and 2%) increased the activities of CAT and PAL by 51% and 16.4%, respectively. The combined application of CaONPs and FM increased the antioxidant defence of mung bean plants as the treatments synergistically improved the functioning of these enzymes. The improved functioning of these enzymes resulted in better crop performance in terms of growth and yield traits. In case of CAT, a least significant difference of 6.477 and in case of PAL the least significant difference of 7.598 was observed among the negative control, positive control, Cd+1% FM group and Cd+2% FM groups (Fig 3C and 3D).

### Gas exchange attributes and total chlorophyll contents of the experimental plants

**Fig 4:**
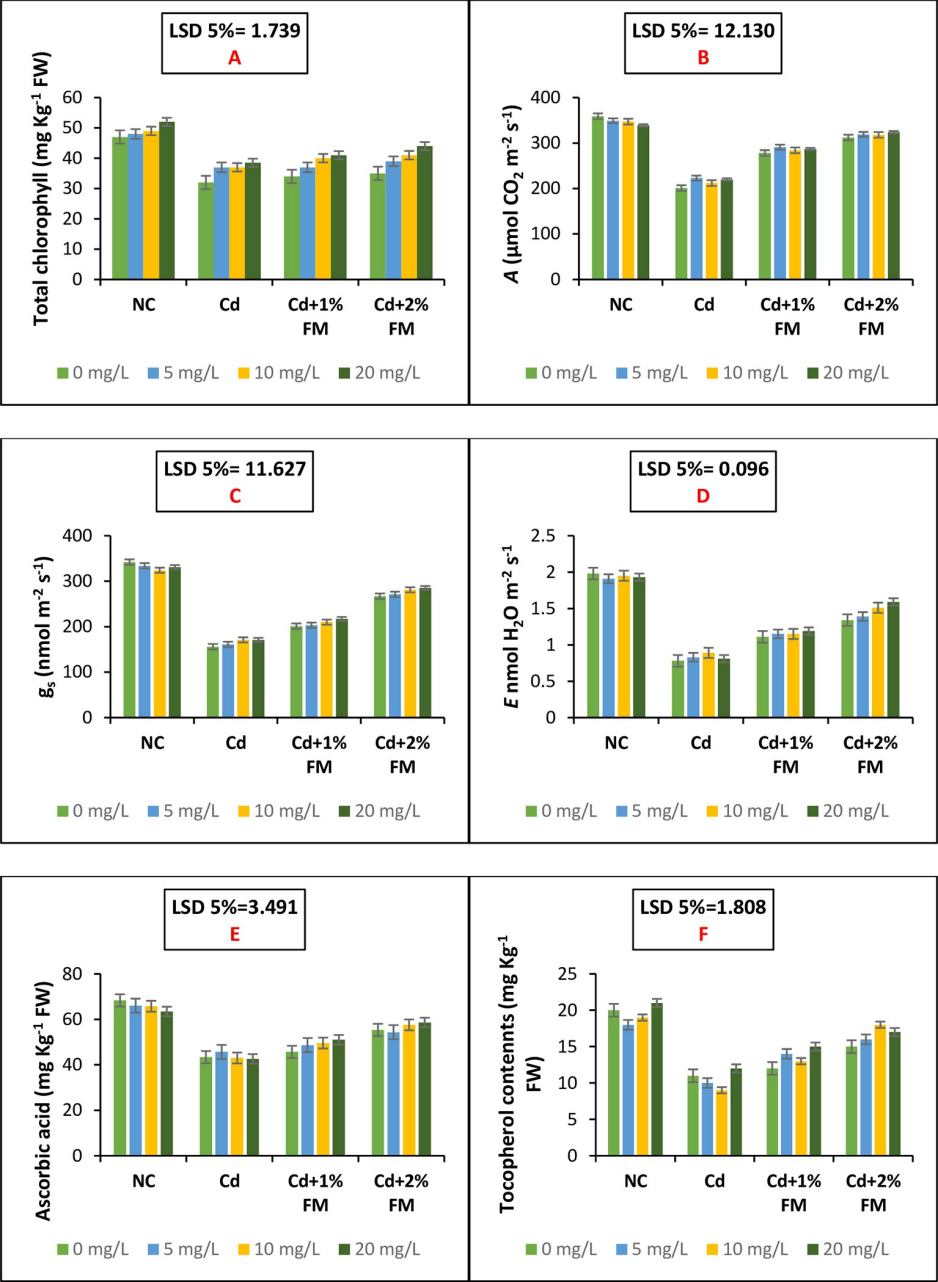
Photosynthetic attributes, gas exchange parameters and shoot vitamin contents of mung bean plants grown under cadmium stress and treated with CaONPs and FM. *A* is CO2 assimilation rate, *E* is net transpiration rate and *gs* is stomata conductance. On the x-axis, NC represents the negative control group; Cd represents the positive control group; Cd+1% FM represents the group treated with Cd and FM 1% and Cd+2% FM represents the group treated with Cd and FM 2%. In the legends 0, 5, 10, and 20 mg/L are root treatment doses of calcium oxide nanoparticles.

Gas exchange parameters and photosynthetic behaviour of the mung bean plants under cadmium stress have been presented in Fig 4. The data in Fig 4A shows that total chlorophyll contents in the mung bean plants and the net photosynthetic rate was reduced upon imposition of cadmium stress. CaONPs improved the leaf net photosynthetic rate (Fig 4B) by improving the total chlorophyll contents. The combined application of CaONPs and FM effectively increased the plant’s photosynthetic ability under cadmium stress. The synergistic dose of 2% FM and 20 mg/L CaONPs increased the total chlorophyll contents by 37.5% and leaf net photosynthetic rate by 61%. The mung bean plants are grown under heavy metal (Cd) stress exhibited poor stomatal conductance (Fig 4C) and transpiration rate (Fig 4D). The decline in the performance of these gas exchange parameters was effectively mitigated by synergistic doses of FM and CaONPs as shown in Fig 4C and 4D. In all cases, the most effective way of pollution reduction was the application of 2% FM as a soil amendment and 20 mg/L CaONPs as root treatment (Table 4). LSD 5% values for stomatal conductance and transpiration rate were calculated as 11.627 and 0.096, respectively which depict a significant statistical difference among the mung plants grown under various treatment groups involving negative control, positive control, Cd +1% FM and Cd+ 2% FM.

**Table 4:** Mean square and p values from the variables studied in the current experiment.

Dependent variable	Treatment effect	Degrees of freedom	Mean Square	P-Value
**Total Tocopherol**	(iii) Columns	3	60.321	0.000***
	(iv) Rows	3	2.834	0.1555 ns
	Error	9	1.271	
**Phenylalanine ammonia lyase**	(iii) Columns	3	881.236	0.020*
	(iv) Rows	3	501.239	0.005**
	Error	9	22.562	
**Catalase**	(iii) Columns	3	1451.765	0.000***
	(iv) Rows	3	94.378	0.0175*
	Error	9	14.403	
**Number of pods per plant**	(iii) Columns	3	36.342	0.014*
	(iv) Rows	3	9.983	0.000***
	Error	9	1.472	
**Pods weight**	(iii) Columns	3	113.245	0.000***
	(iv) Rows	3	157.987	0.000***
	Error	9	9.117	
**Seed count per pod**	(iii) Columns	3	7.473	0.003**
	(iv) Rows	3	4.474	0.000***
	Error	9	0.445	
**Thousand seeds weight**	(iii) Columns	3	32.089	0.000***
	(iv) Rows	3	14.252	0.000***
	Error	9	0.753	
**Malondialdehyde**	(iii) Columns	3	2234.343	0.000***
	(iv) Rows	3	731.722	0.000***
	Error	9	46.452	
**Hydrogen peroxide**	(iii) Columns	3	649.128	0.000***
	(iv) Rows	3	407.124	0.000***
	Error	9	29.102	

*, ** and *** = significant at 0.05, 0.01, and 0.001 levels, respectively. ns; non-significant ranges

### Activities of non-enymatic antioxidants

Shoot vitamin (Vitamin C and E) contents of mung bean plants declined upon imposition of cadmium stress. The levels of ascorbic acid (Vitamin C) and total tocopherol contents (Vitamin K) were improved significantly (LSD 3.491 and 1.808, respectively) in all instances by the root treatment CaONPs (Fig 4E and 4F). The application of FM under CaONPs treatment was effective in mitigating cadmium stress and improving plant performance. About 2% FM and 20 mg/L CaONPs treatment increased ascorbic acid contents by 33% and tocopherol contents by 54% and proved the best treatment in increasing shoot vitamin contents (Fig 4E and 4F).

### Yield attributes of the experimental plants as influenced by CaONPs and FM treatment under cadmium stress

**Fig 5:**
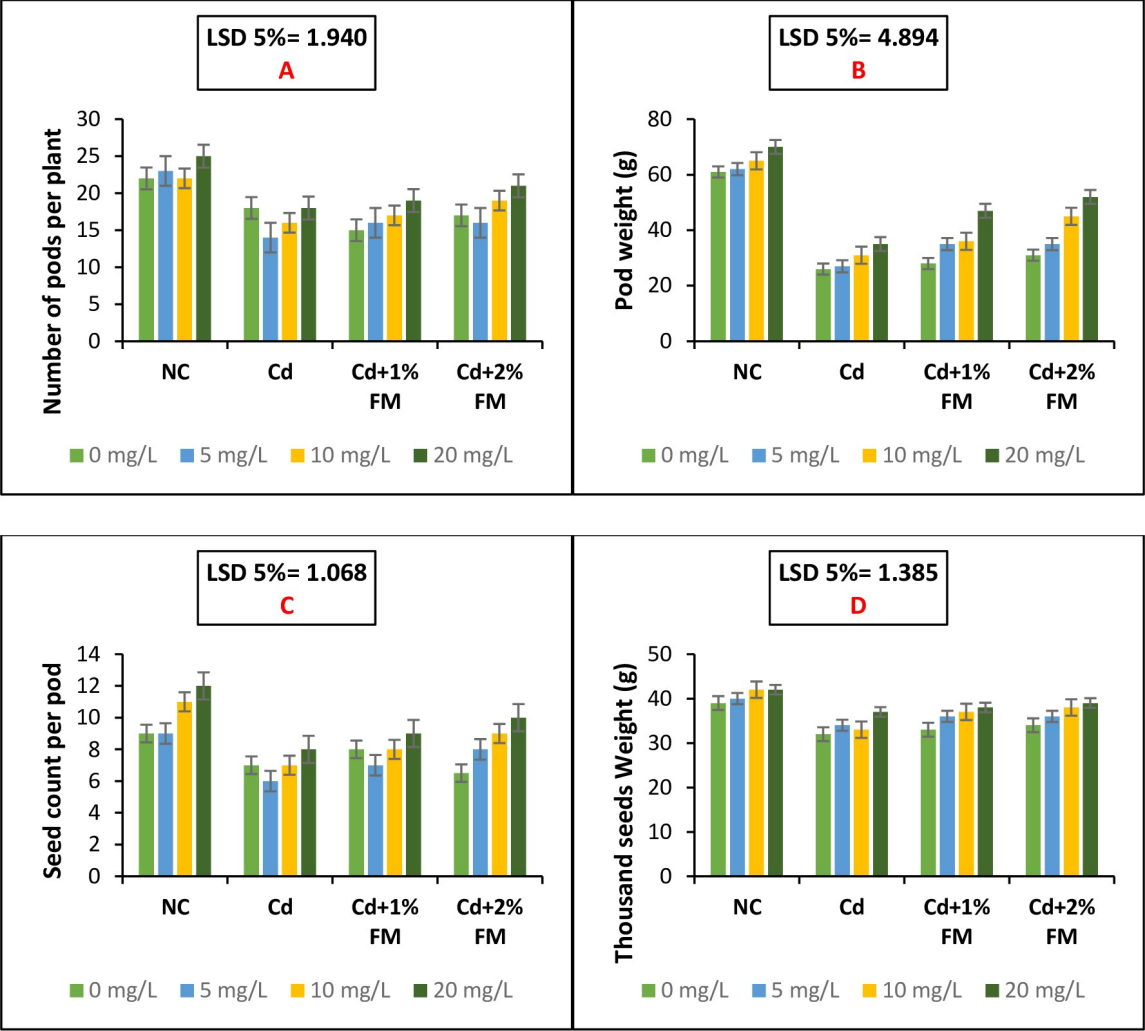
Yield attributes of mung bean plants grown under cadmium stress and treated with CaONPs and FM. On the x-axis, NC represents the negative control group; Cd represents the positive control group; Cd+1% FM represents the group treated with Cd and FM 1% and Cd+2% FM represents the group treated with Cd and FM 2%. In the legends 0, 5, 10, and 20 mg/L are root treatment doses of calcium oxide nanoparticles.

The yield traits of mung bean plants were recorded in the present experiment. The data presented in the Fig 5 shows that yield of mung beans declines under cadmium stress. Combined treatment with CaONPs and FM effectively ameliorates the hazardous impacts of cadmium toxicity and improves crop performance in terms of yield. A least significance difference of 1.068 and 1.385 was observed among the treatment groups negative control, positive control, Cd +1% FM and Cd+ 2% FM for seed count per pod and thousand seed weight respectively. The studied yield attributes included the number of pods per plant (Fig 5A), pods weight (Fig 5B), the number of seeds per pod (Fig 5C), and thousand seeds weights (Fig 5D). The weight of thousand seeds was also recorded. All the mentioned parameters were improved significantly upon treatment with CaONPs and FM under cadmium stress. CaONPs at 20 mg/L and 2% FM increased number of pods per plant by 16.66%, seed count per pod by 42.85% and thousand seeds weights by 22%. A least significance difference of 1.940 and 4.894 was observed among the treatment groups negative control, positive control, Cd +1% FM and Cd+ 2% FM for number of pods per plant and pod weights per plant, respectively.

### Leaf calcium and relative water contents of the mung bean plants

Leaf relative water contents and dry leaf Ca^+2^ was decreased significantly upon treatment with Cd salt. Root treatments with calcium oxide nanoparticles and farmyard manure application to the soil raised leaf relative water contents and leaf calcium contents. Overall, the 2% FM treatment along with 20 mg/L CaONPs proved among the best of the treatments in alleviating Cd toxicity (Table 5).

**Table 5:** Leaf Ca^+2^ and leaf relative water contents of mung bean plants followed in the study.

Parameters	Calcium oxide Nanoparticles (mg/L)	Negative Controls	Cadmium (150 uM)	Cd +1% FM	Cd+2% FM
Leaf relative water contents (%)	0	73± 1.4	58±1.45	65± 1.5	67± 1.64
	5	76± 1.5	61±1.54	69± 1.43	72±1.3
	10	81± 1.9	64± 1.21	71± 2	75± 2.1
	20	83± 2	68± 1.31	73± 1.83	79± 1.91
Leaf calcium contents (mg/g Dry weight)	0	4.87± 0.07	1.91± 0.07	2.78± 0.04	3.87± 0.07
	5	5.21± 0.08	2.31± 0.06	3.24± 0.05	4.13± 0.07
	10	5.37± 0.95	2.97± 0.03	3.86± 0.06	4.15± 0.05
	20	5.59± 0.05	3.43± 0.04	3.9± 0.05	4.31± 0.07

### Correlation analysis, heat map and principal component analysis of the variables studied in the experiment

**Fig 6:**
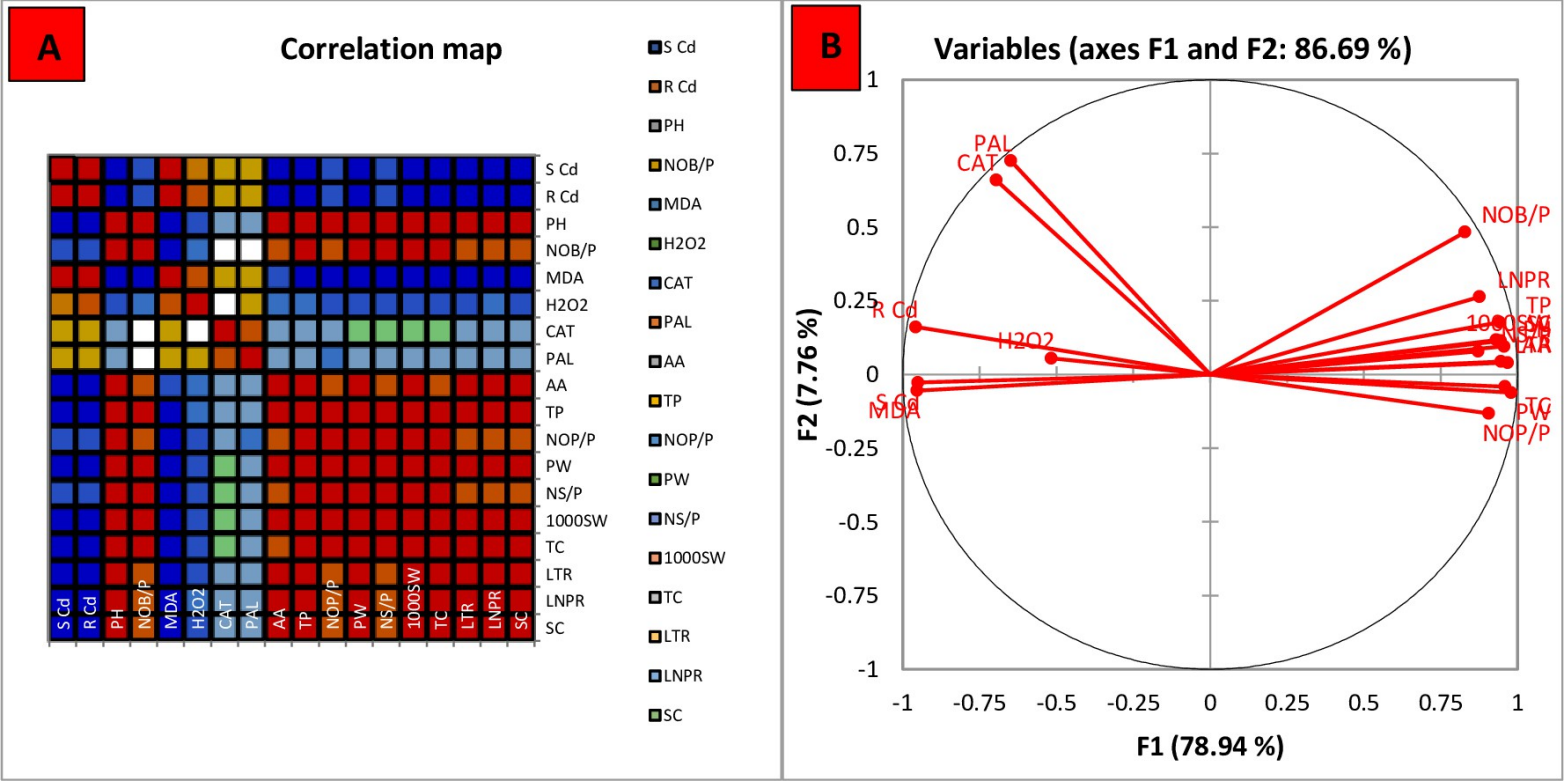
(A). Spearman correlation map among the studied variables (B) Principal component analysis wagon wheel. S Cd: Shoot cadmium contents; R Cd: Root cadmium contents; PH: Plant height; NOB/P: Number of branches per plant; MDA: malondialdehyde contents; H2O2: Hydrogen peroxide levels; CAT: Catalase functioning; PAL: Phenylalanine ammonia lyase activity; AA: Ascorbic acid; TP: Total Tocopherol contents; NOP/P: Number of pods per plant; PW: Pod weight; NS/P: number of seeds per pod; 1000 SW: Thousand seeds weight; TC: Total Chlorophyll contents.

The Correlation analysis evaluates the statistical relationship between two variables. When one variable rises and the other one follows, there is a positive link between the two variables. When there is a reduction in one variable, there will be an increase in the other, and vice versa, in a negative correlation. Spearman correlation matrix was constructed to comprehend the results of the present study and to understand the correlation among the variables in mitigation of Cd stress (Table 6; Fig 6A). There was a negative significant correlation between shoot and root cadmium contents with the height and number of branches of mung bean plants. A positive significant correlation was observed between the decreasing cadmium contents in roots and shoots and decreasing osmotic stress indicators. Furthermore, a negative but significant correlation was observed between the cadmium levels and yield trends of mung bean plants. The data presented in the Fig 6B shows the principal components analysis (PCA) wagon wheel. It is clear that two factors F1 and F2 contributed 78.94% and 7.76% in defining the variance. PCA loading charts show that there is a significant correlation among the variables.

**Table 6:** Spearman correlation matrix for the variables of mung bean plants followed in the current experiment.

Variables	S Cd	R Cd	PH	NOB/P	MDA	H2O2	AA	TP	NOP/P	PW	NS/P	1000SW	TC	LTR	LNPR
S Cd	1														
R Cd	0.955*														
PH	-0.908*	-0.911*													
NOB/P	-0.768*	-0.753*	0.875*												
MDA	0.841*	0.832*	-0.970*	-0.852*											
H2O2	0.582*	0.620*	-0.719*	-0.590*	0.747*										
AA	-0.928*	-0.977*	0.869*	0.731*	-0.793*	-0.513*									
TP	-0.934*	-0.941*	0.941*	0.822*	-0.878*	-0.533*	0.934*								
NOP/P	-0.714*	-0.729*	0.860*	0.686*	-0.881*	-0.618*	0.744*	0.808*							
PW	-0.856*	-0.854*	0.963*	0.867*	-0.990*	-0.773*	0.816*	0.882*	0.861*						
NS/P	-0.729*	-0.741*	0.881*	0.834*	-0.908*	-0.708*	0.703*	0.817*	0.835*	0.904*					
1000SW	-0.823*	-0.821*	0.950*	0.860*	-0.983*	-0.688*	0.804*	0.868*	0.854*	0.977*	0.885*				
TC	-0.823*	-0.832*	0.947*	0.848*	-0.966*	-0.792*	0.797*	0.844*	0.830*	0.973*	0.886*	0.974*			
LTR	-0.946*	-0.987*	0.915*	0.784*	-0.844*	-0.631*	0.970*	0.954*	0.729*	0.867*	0.767*	0.834*	0.840*		
LNPR	-0.964*	-0.973*	0.882*	0.736*	-0.817*	-0.529*	0.969*	0.941*	0.692*	0.834*	0.705*	0.821*	0.807*	0.974*	
SC	-0.972*	-0.989*	0.931*	0.773*	-0.869*	-0.624*	0.966*	0.956*	0.770*	0.882*	0.776*	0.849*	0.849*	0.982*	0.975*

Values with * are significantly correlated at 5% S Cd: Shoot cadmium contents; R Cd: Root cadmium contents; PH: Plant height; NOB/P: Number of branches per plant; MDA: malondialdehyde contents; H_2_O_2_: Hydrogen peroxide level; AA: Ascorbic acid; TP: Total Tocopherol contents; NOP/P: Number of pods per plant; PW: Pod weight; NS/P: number of seeds per pod; 1000 SW: Thousand seeds weight; TC: Total Chlorophyll contents

**Fig 7:**
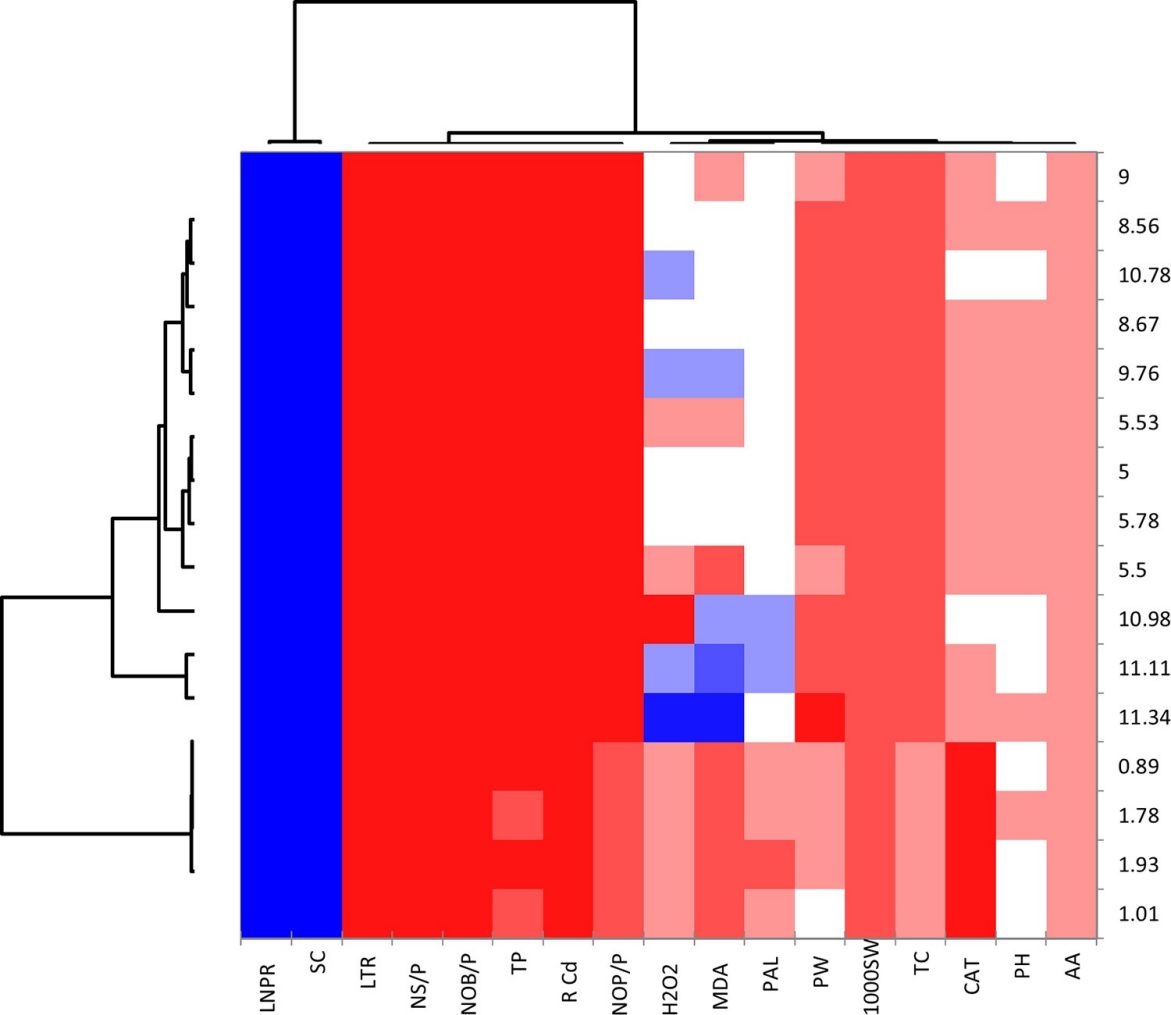
Heat map loading charts on the data of various variables followed in the study. S Cd: Shoot cadmium contents; R Cd: Root cadmium contents; PH: Plant height; NOB/P: Number of branches per plant; MDA: malondialdehyde contents; H_2_O_2_: Hydrogen peroxide level; AA: Ascorbic acid; TP: Total Tocopherol contents; NOP/P: Number of pods per plant; PW: Pod weight; NS/P: number of seeds per pod; 1000 SW: Thousand seeds weight; TC: Total Chlorophyll contents.

The Fig 7 describes a clustered heat map that represents the effect of Cd stress on mung bean plants and the impact of treatments with FM and CaONPs. Higher concentrations of CaONPs and FM indicate a significant reduction in levels of root and shoot cadmium contents and osmotic stress indicators. It is noteworthy that root treatment with various levels of CaONPs and soil amendment with FM effectively moderated the cadmium stress and improved the growth and yield performance of the plant.

## Discussion

Plants, particularly crops have been facing many biotic and abiotic stresses in the environment. The crops are of paramount importance because these plants provide staple and basic food necessities for the people around the world. The crops are being imposed drastic decreasing impacts due to facing severe issues which are drastically reducing the yield and quality of the food [25]. Similarly, mung bean crop is facing environmental stress of Cd and its yield is being decreasing and quality is also deteriorating. Therefore, there is increasing need of crop friendly and bio-rationale agricultural practices for sustainable agriculture.

Calcium nutrient is not only a nutrient rather it is an important signalling molecule. Calcium deficiency leads to more susceptibility to the environmental stresses faced by the plants. Furthermore, the application of organic supplements such as FM has been practiced since the emergence of agriculture and its efficacy to the agricultural soil has been reported by several researchers [11, 14]. Therefore, in present study, the FM and supplementation in the form of CaONPs was practiced.

Plants use a variety of defence mechanisms while under Cd stress to lessen its negative effects. The major functions of osmolytes are to lower water potential, keep water balance, safeguard intracellular structures, and lessen oxidative damage. Under stressful circumstances, plants increase organic osmotic pressure to maintain tissue water content and to upregulate antioxidant activity. When exposed to oxidative stress brought on by harmful heavy metals, amino acids operate as organic osmolytes, take part in osmoregulation, stabilise proteins in membranes, maintain ionic homeostasis, scavenge ROS and moderate redox potential.

Calcium ions (Ca^+2^) and Cadmium ions (Cd^+2^) have the same valence and almost the same ionic radii. The ionic radius of calcium is 9.9x10^-2^ nm and that of cadmium is 9.7x10^-2^ nm. This is the reason why the application of Ca reduced Cd acquisition by roots and shoots of mung bean plants [26]. Both Cd^+2^ and Ca^+2^ compete for cation exchange sites available on clay minerals and soil organic matter. Thus, the availability of Ca to the Cd polluted soils reduces Cd acquisition. Ca^+2^ not only reduces the Cd^+2^ uptake rather it continues to be translocate by the membrane calcium channels causing normal growth and metabolism [14]. The application of FM further enhanced tolerance to Cd toxicity. The application of FM has binding effects on heavy metals. The FM contains humic acid which can bind to heavy metals such as Cd reducing their bioavailability to the plants. Additionally, the humic acid improves nutrient acquisition patterns by improving root architecture and mobilisation of nutrient around the rhizosphere, so it can be assumed that the application of CaONPs and FM rejuvenated the Cd-contaminated soil resulting in nutrient mobilisation and reduced Cd uptake. This overall effect might be responsible for improved performance of mug bean plants under cadmium stress [27].

The growth of mung bean plants in terms of the number of branches and plant height was improved by the application of CaONPs and FM. Hosseini *et al*., [28] reported that calcium application and bioavailability as Ca^+2^ increased the biosynthesis of putrescine. The putrescine modulates the growth and biomass of mung bean plants. The application of calcium nanoparticles to the soil promotes nutrient acquisition. The uptake of nutrients such as Si and Mg is essential to the accumulation of sugars and osmolytes which ultimately improve plant biomass. Furthermore, FM is a valuable source of several micronutrients and macronutrients such as NPK which are essential for normal growth and metabolism. In literature, the application of FM has been reported to successfully mitigate the toxicity of several heavy metals such as Al, As, Cd, and Pd. Application of FM leads to improvement in soil physical and chemical characteristics which are important for proper growth and metabolism [29, 30].

Total photosynthesis and chlorophyll contents of mung bean plants were improved by the FM and CaONPs application. This is due to calcium regulating the NAD^+^ kinase enzyme of the chloroplast. Calcium as a mineral is important to maintain the envelope structure of plastids and as a signalling molecule promotes the activity of photosystem-II [30]. This overall effect led to the better photosynthetic potential of mung bean plants. The gas exchange parameters such as net transpiration rate and stomata conductance were improved significantly upon fertilizing the soil with CaONPs nanoparticles and FM. Increased protein synthesis in the current study, increased ROS detoxification as shown by increased antioxidant defence, and decreased levels of the osmotic stress markers MDA and hydrogen peroxide are just a few examples of how nanoparticles improve stress tolerance in plants by improving water uptake and showing differential abundance of proteins involved in oxidation-reduction. Another potential reason for the improved behaviour of mung bean plants in terms of plant water relations may be greater translocation of shoot minerals, such as calcium uptake. Improved structure and porosity of the soil due to the addition of FM results in better uptake of water. This overall effect leads to better transpiration and stomatal conductance [31].

Responding to the environment is required for normal homeostasis and for plant adaptations to detrimental conditions. FM and CaONPs amended soil reduced Cd uptake by the root and shoots of mung bean plants and improved the yield of mung bean plants in terms of increased pod number, pod weights, seed count, and seed weights. The improved yield traits are due to FM application which leads to better nutrient availability and acquisition culminating in a good yield. FM application leads to better growth of plants since it stimulates the growth of beneficial microbes in the rhizosphere [32]. These beneficial microbes release proteins into the soil which are potentially ameliorating in cadmium-stressed soils. Additionally, the decomposition of FM in the soil releases organic acids essential to proper growth and homeostasis [14]. CaONPs supplementation stimulates better nutrient acquisition patterns in plants and thus overall yield is improved. Furthermore, the decrease in lipid peroxidation in our results by FM and CaONPs supplementation indicates the protection of biological membranes. Ca as a mineral stabilizes the structure of the biological membrane by binding to the phospholipids in the membrane. Membrane stability is crucial to the maintenance of ion channels and nutrient acquisition by plants [33].

Cytosolic non-enzymatic antioxidants were studied in terms of leaf ascorbic acid (vitamin C) and total tocopherol contents (vitamin E). In our results, exposure to Cd stress resulted in decreased ascorbate and tocopherols accumulation compared to negative controls. This might be due to accumulation of the heavy metal in the roots of mug bean plants. The accumulation of Cd in the roots has an inhibitory effect on the ions uptake, mineral nutrition and normal metabolism which may lead to decreased cytosolic antioxidant such as tocopherols, ascorbate, glutathione and carotenoids. The application of calcium nanoparticles and FM improved the contents of these vitamins. The ascorbic acid is a key substrate for detoxification of ROS [34] and an important oxidant scavenger thus its accumulation under Cd stress leads to activation of the stress tolerance mechanism. Additionally, the tocopherol enhancement leads to a stress moderation mechanism in plants since tocopherols are involved in protecting plants from oxygen toxicity. An increase in the contents of these metabolites might be due to the role of CaONPs and FM in increasing the nutrient acquisition pattern by the mung plants [35]. FM is a source of several mineral including sulphur which is crucial to biosynthesis of cytosolic antioxidants. It can be assumed that application of FM might have provided certain minerals required for biosynthesis of antioxidants.

In the present research, observations were recorded on important antioxidant enzymes such as catalase (CAT) and phenylalanine ammonium lyase (PAL). In plants, CAT is a major scavenger of hydrogen peroxide, a form of reactive oxygen species (ROS) [36]. In our results, we noted that supplementing the soil with FM and CaONPs leads to enhancement in the activity of catalase. Furthermore, we noted that levels of hydrogen peroxide were found to decline upon treatment with CaONPs and FM. Studies have reported that the application of calcium leads to the activation of defence genes in plants which are responsible for up-regulating the functioning of antioxidants. PAL is an important enzyme involved in the phenylpropanoid pathway. PAL is a key enzyme in dealing with environmental stressors in plants [37]. An increase in PAL activities might be due to Cd toxicity-induced cellular damage as an increase in the functioning of these enzymes is a part of the internal defence response by the plants. Furthermore, the activation of phenylpropanoid pathways is key to the synthesis of various phytochemicals and botanicals which might help in stress alleviation [38, 39]. PAL activity depends on its expression from the several PAL encoding genes in plants. The expression of these genes is epigenetically controlled by environmental signals such as abiotic and heavy metal stress. Several studies have reported the impact of organic manures in boosting antioxidant defence [40, 41]. Application of CaONPs and FM proved better in alleviating cadmium toxicity faced by the mung bean plants. Keeping in view the climate change mediated food insecurity the treatments might be useful in increasing growth and production of pulse crops and thus are helpful in sustainable agriculture.

Under conditions of heavy metal stress, calcium is a crucial component for plant osmotic adaptation. A universal messenger, calcium is known to control metabolic functions, serve as a converter, control photosynthesis, and maintain healthy levels of vital nutrients. Under heavy metal stress, it can modulate a wide range of essential biological functions, including growth, physiology, and metabolic activities. Through improved growth, physiochemical, and genetic methods, additional work may be done to comprehend the specific pathways of Ca-induced Cd stress alleviation.

## Conclusions

The present work deciphered FM and CaONPs mediated better physiological and biochemical behaviour of mung bean plants under cadmium toxicity. Soil supplementation with Ca and FM improved the activities of antioxidant enzymes and shoot vitamin contents of mung bean plants which is crucial to induction of tolerance against abiotic stress. Net photosynthesis, stomata conductance and total chlorophyll contents improved and Cd toxicity was moderated. Calcium as a metal has prime significance due to its signalling role and the addition of FM to the soil has proven in modulating soil biological activities. On the basis of our results, we recommend the use of FM and CaONPs supplementation to the croplands affected by the stress of the heavy metals. However, the author recommends further exploration under field conditions and in terms of other food crops to further validate the results.

## References

[1] Dahiya PK, Linnemann AR, Van Boekel MA, Khetarpaul N, Grewal RB, Nout MJ. Mung bean: Technological and nutritional potential. Critical reviews in food science and nutrition. 2015 Apr 16;55(5):670–88. doi: 10.1080/10408398.2012.671202 24915360

[2] Mao F, Nan G, Cao M, Gao Y, Guo L, Meng X, et al. The metal distribution and the change of physiological and biochemical process in soybean and mung bean plants under heavy metal stress. International journal of phytoremediation. 2018 Sep 19;20(11):1113–20. doi: 10.1080/15226514.2017.1365346 30156914

[3] El Rasafi T, Oukarroum A, Haddioui A, Song H, Kwon EE, Bolan N, et al. Cadmium stress in plants: A critical review of the effects, mechanisms, and tolerance strategies. Critical Reviews in Environmental Science and Technology. 2022 Mar 4;52(5):675–726.

[4] Shanmugaraj BM, Malla A, Ramalingam S. Cadmium stress and toxicity in plants: an overview. Cadmium toxicity and tolerance in plants. 2019 Jan 1:1–7.

[5] Liu L., Gao H., Li S., Han Z. and Li B., 2021. Calcium signaling networks mediate nitrate sensing and responses in Arabidopsis. Plant Signaling & Behavior, 16(10), p.1938441. doi: 10.1080/15592324.2021.1938441 34180337 PMC8330996

[6] Thor K. Calcium—nutrient and messenger. Frontiers in plant science. 2019 Apr 25;10:440. doi: 10.3389/fpls.2019.00440 31073302 PMC6495005

[7] Gandhi N, Shruthi Y, Sirisha G, Anusha CR. Facile and eco-friendly method for synthesis of calcium oxide (CaO) nanoparticles and its potential application in agriculture. Saudi Journal of Life Sciences, 2021;6:89–103.

[8] Meier S, Moore F, Morales A, González ME, Seguel A, Meriño-Gergichevich C, et al. Synthesis of calcium borate nanoparticles and its use as a potential foliar fertilizer in lettuce (Lactuca sativa) and zucchini (Cucurbita pepo). Plant physiology and biochemistry. 2020 Jun 1;151:673–80. doi: 10.1016/j.plaphy.2020.04.025 32353673

[9] Syu CH, Yu CH, Lee DY. Effect of applying calcium peroxide on the accumulation of arsenic in rice plants grown in arsenic-elevated paddy soils. Environmental Pollution. 2020 Nov 1;266:115140. doi: 10.1016/j.envpol.2020.115140 32653722

[10] Ma Q, Wen Y, Wang D, Sun X, Hill PW, Macdonald A, et al. Farmyard manure applications stimulate soil carbon and nitrogen cycling by boosting microbial biomass rather than changing its community composition. Soil Biology and Biochemistry. 2020 May 1;144:107760.

[11] Singh L, Sukul P. Impact of vermicompost, farmyard manure, fly ash and inorganic fertilizers on growth and yield attributing characters of maize (*Zea mays* L.). Plant Arch. 2019;19(2):2193–200.

[12] Dewis J, Freitas F. Physical and chemical methods of soil and water analysis. FAO soils Bulletin. 1970(10).

[13] Waqas Mazhar M, Ishtiaq M, Hussain I, Parveen A, Hayat Bhatti K, Azeem M, et al. Seed nano-priming with Zinc Oxide nanoparticles in rice mitigates drought and enhances agronomic profile. PLOS ONE. 2022 Mar 24;17(3):e0264967. doi: 10.1371/journal.pone.0264967 35324949 PMC8947021

[14] Khan K.A., Shoaib A., Arshad Awan Z., Basit A. and Hussain M., 2018. Macrophomina phaseolina alters the biochemical pathway in Vigna radiata chastened by Zn2+ and FYM to improve plant growth. Journal of Plant Interactions, 13(1), pp.131–140.

[15] Ahmed N, Ahsen S, Ali MA, Hussain MB, Hussain SB, Rasheed MK, et al. Rhizobacteria and silicon synergy modulates the growth, nutrition and yield of mungbean under saline soil. Pak. J. Bot. 2020 Feb 1;52(1):9–15.

[16] Cakmak I, Horst WJ. Effect of aluminium on lipid peroxidation, superoxide dismutase, catalase, and peroxidase activities in root tips of soybean (Glycine max). Physiologia plantarum. 1991 Nov;83(3):463–8.

[17] Velikova V, Yordanov I, Edreva A. Oxidative stress and some antioxidant systems in acid rain-treated bean plants: protective role of exogenous polyamines. Plant science. 2000 Feb 7;151(1):59–66.

[18] Chance B, Maehly AC. [136] Assay of catalases and peroxidases. 10.1002/9780470110171.ch14 13193536

[19] Kim DS, Hwang BK. An important role of the pepper phenylalanine ammonia-lyase gene (PAL1) in salicylic acid-dependent signalling of the defence response to microbial pathogens. Journal of experimental botany. 2014 Jun 1;65(9):2295–306. doi: 10.1093/jxb/eru109 24642849 PMC4036500

[20] Arnon DI. Copper enzymes in isolated chloroplasts. Polyphenoloxidase in Beta vulgaris. Plant physiology. 1949 Jan;24(1):1. doi: 10.1104/pp.24.1.1 16654194 PMC437905

[21] Mukherjee SP, Choudhuri MA. Implications of water stress‐induced changes in the levels of endogenous ascorbic acid and hydrogen peroxide in Vigna seedlings. Physiologia plantarum. 1983 Jun;58(2):166–70.

[22] Baker H. Plasma tocopherol in man at various times after ingesting free or acetylated tocopherol.

[23] Shehzad F, Ali Q, Ali S, Al-Misned FA, Maqbool S. Fertigation with Zn-Lysine Confers Better Photosynthetic Efficiency and Yield in Water Stressed Maize: Water Relations, Antioxidative Defense Mechanism and Nutrient Acquisition. Plants. 2022 Feb 1;11(3):404. doi: 10.3390/plants11030404 35161385 PMC8838349

[24] Abbas T, Rizwan M, Ali S, Zia-ur-Rehman M, Qayyum MF, Abbas F, et al. Effect of biochar on cadmium bioavailability and uptake in wheat (Triticum aestivum L.) grown in a soil with aged contamination. Ecotoxicology and environmental safety. 2017 Jun 1;140:37–47. doi: 10.1016/j.ecoenv.2017.02.028 28231504

[25] Waqas Mazhar M, Ishtiaq M, Maqbool M, Akram R, Shahid A, Shokralla S, et al. Seed Priming with Iron Oxide Nanoparticles Raises Biomass Production and Agronomic Profile of Water-Stressed Flax Plants. Agronomy. 2022 Apr 19;12(5):982.

[26] Huang D, Gong X, Liu Y, Zeng G, Lai C, Bashir H, et al. Effects of calcium at toxic concentrations of cadmium in plants. Planta. 2017 May;245(5):863–73. doi: 10.1007/s00425-017-2664-1 28204874

[27] Irfan M, Mudassir M, Khan MJ, Dawar KM, Muhammad D, Mian IA, et al. Heavy metals immobilization and improvement in maize (Zea mays L.) growth amended with biochar and compost. Scientific Reports. 2021 Sep 16;11(1):1–9. 34531439 10.1038/s41598-021-97525-8 PMC8446096

[28] Hosseini SA, Réthoré E, Pluchon S, Ali N, Billiot B, Yvin JC. Calcium application enhances drought stress tolerance in sugar beet and promotes plant biomass and beetroot sucrose concentration. International journal of molecular sciences. 2019 Aug 2;20(15):3777. doi: 10.3390/ijms20153777 31382384 PMC6696248

[29] Mbarki S, Skalicky M, Talbi O, Chakraborty A, Hnilicka F, Hejnak V, et al. Performance of Medicago sativa grown in clay soil favored by compost or farmyard manure to mitigate salt stress. Agronomy. 2020 Jan 9;10(1):94.

[30] Azeez L, Lateef A, Adetoro RO, Adeleke AE. Responses of Moringa oleifera to alteration in soil properties induced by calcium nanoparticles (CaNPs) on mineral absorption, physiological indices and photosynthetic indicators. Beni-Suef University Journal of Basic and Applied Sciences. 2021 Dec;10(1):1–5.

[31] Pirayesh N, Giridhar M, Khedher AB, Vothknecht UC, Chigri F. Organellar calcium signaling in plants: An update. biochimica et biophysica Acta (bbA)-molecular Cell Research. 2021 Apr 1;1868(4):118948. 10.1016/j.bbamcr.2021.118948 33421535

[32] Bashir A, Rizwan M, ur Rehman MZ, Zubair M, Riaz M, Qayyum MF, et al. Application of co-composted farm manure and biochar increased the wheat growth and decreased cadmium accumulation in plants under different water regimes. Chemosphere. 2020 May 1;246:125809. doi: 10.1016/j.chemosphere.2019.125809 31927378

[33] Aldon D, Mbengue M, Mazars C, Galaud JP. Calcium signalling in plant biotic interactions. International journal of molecular sciences. 2018 Feb 27;19(3):665. doi: 10.3390/ijms19030665 29495448 PMC5877526

[34] Wang J, Huang R. Modulation of ethylene and ascorbic acid on reactive oxygen species scavenging in plant salt response. Frontiers in plant science. 2019 Mar 18;10:319. doi: 10.3389/fpls.2019.00319 30936887 PMC6431634

[35] Sadiq M, Akram NA, Ashraf M, Al-Qurainy F, Ahmad P. Alpha-tocopherol-induced regulation of growth and metabolism in plants under non-stress and stress conditions. Journal of Plant Growth Regulation. 2019 Dec;38(4):1325–40.

[36] Tehrani HS, Moosavi-Movahedi AA. Catalase and its mysteries. Progress in Biophysics and Molecular Biology. 2018 Dec 1;140:5–12. doi: 10.1016/j.pbiomolbio.2018.03.001 29530789

[37] Barros J, Dixon RA. Plant phenylalanine/tyrosine ammonia-lyases. Trends in plant science. 2020 Jan 1;25(1):66–79. doi: 10.1016/j.tplants.2019.09.011 31679994

[38] Levy HL, Sarkissian CN, Scriver CR. Phenylalanine ammonia lyase (PAL): From discovery to enzyme substitution therapy for phenylketonuria. Molecular Genetics and Metabolism. 2018 Aug 1;124(4):223–9. doi: 10.1016/j.ymgme.2018.06.002 29941359

[39] Varga A, Csuka P, Sonesouphap O, Bánóczi G, Toşa MI, Katona G, et al. A novel phenylalanine ammonia-lyase from Pseudozyma antarctica for stereoselective biotransformations of unnatural amino acids. Catalysis Today. 2021 Apr 15;366:185–94.

[40] ur Rehman MZ, Rizwan M, Khalid H, Ali S, Naeem A, Yousaf B, et al. Farmyard manure alone and combined with immobilizing amendments reduced cadmium accumulation in wheat and rice grains grown in field irrigated with raw effluents. Chemosphere. 2018 May 1;199:468–76. doi: 10.1016/j.chemosphere.2018.02.030 29454169

[41] Chahal HS, Singh A, Dhillon IS, Kaur J. Farmyard manure: A boon for integrated nutrient management. International Journal of Agriculture, Environment and Biotechnology. 2020 Dec 1;13(4):483–95.

